# Bright's Disease and Life Insurance

**Published:** 1890-08-09

**Authors:** 


### The Doctor's Pulpit.

### BRIGHT'S DISEASE AND LIFE INSURANCE.

The "passing of the doctor" for life insurance is one of those ordeals which most nervous people dread, and which deters not a few from insuring their lives altogether. In quite recent times it has been found necessary by medical men to make very careful investigation into the state of the kidneys on account of the prevalence of what has long been known as Bright's Disease. The name Bright's Disease is of very wide significance in medicine, and is used so loosely that in a careless doctor's mouth it may mean sometimes a temporary symptom of very small significance, and at other times such a degree of structural disorganisation of the kidney as must inevitably terminate in death within a period to be measured by weeks or days. The intelligent medical man, being familiar with this loose use of the term, measures the significance of any particular case of Bright's Disease by its peculiar qualifying adjective. It may be a case of acute or of chronic Bright; of waxy, or of fatty, or of cirrhotic Bright; or it may be a case of simple temporary albuminuria, due to ascertainable and removable causes, and of no more serious import than an attack of diarrhœa. But, though the medical man is familiar with all this, the unfortunate layman may be entirely ignorant of it; and if, on making a proposal to an insurance office, he should fall into the hands of a careless or unscientific medical examiner, he may be placed in the position of a rejected and unhappy man for the remainder of his life. So recently as five-and-twenty years ago almost every physician in the world would have pronounced against any proposer who had the slightest trace of albumin in his urine. If such a proposer had inquired as to the cause of his rejection, he would have been informed that he was suffering from Bright's Disease, or, at least, that he was threatened with it; and supposing him to have been of a nervous disposition, the rest of his days would have been passed in fear and trembling.

Doctors know better nowadays; or, at any rate, the best of them do. Thanks to Sir Andrew Clark in England, and to many other British and Continental physicians, Bright's Disease is intelligently understood; or, rather, the various diseases to which the kidney is subject, are quite familiar in their history and details to competent medical men; and their significance is correctly gauged, both at the insurance office and in the consulting-room. One of the most competent of Continental physicians is Dr. Semmola, a member of the Italian Senate, and distinguished pupil of Claude Bernard. Last week Dr. Semmola read a paper at the Paris Academy of Medicine on Bright's Disease; and the conclusions which he there set forth ought to find their way, not only into every insurance office and medical consulting-room, but also into that common store of knowledge which is at the daily service of every moderately educated man. Dr. Semmola showed that one variety of Bright's Disease, simple albuminuria, could be artificially induced in healthy animals by the subcutaneous injection of white of egg, with certain metallic salts. He further stated that persons who often ate eggs in various forms and did not take a good deal of exercise might have albuminuria of a comparatively harmless kind. Such persons, he said, need be under no alarm unless their symptoms were accompanied by positive kidney inflammation. He affirmed, also—and this it is well to bear in mind—that juicy meats and eggs in excessive quantities had a tendency to the production of kidney disease. He had, he said, been studying Bright's Disease for the last forty years, and had come to the conclusion that it very frequently arose from the eating of too much rich, nitrogenous food. His advice to all sorts and conditions of men and women was that they should be strictly temperate in eating, as well as in drinking, if they wished to preserve the purity of their blood and to avoid diseases of the kidneys.

The question of Life Assurance is so important from the point of view of provision for bereaved widows and families that every hindrance should be removed from the path of potential insurers, and every facility and encouragement should be offered to them. Most of the life offices are now aware of the progress that has been made in medical knowledge, and of the more scientific and common-sense views that prevail among the better educated members of the medical profession; but the public generally are almost in as profound a state of ignorance as they were two or three hundred years ago. This is greatly to be deplored.

It is difficult to see how a more intelligent condition of the public mind can be brought about unless medical men will themselves take the initiative in the instruction of their patients. Unhappily between the ideas that are prevalent in the medical profession and those that are current in the minds of the lay public there is a great gulf fixed, so that for all practical purposes, except those of the physician and the surgeon, scientific medical knowledge might as well be in another planet. This is a very serious loss to the working resources of civilisation; and in no department of life is it more injurious than in its effects on Life Assurance. Of all persons medical men could do the most to dissipate the general ignorance and prejudice that operate to deter men from approaching the doors of insurance offices. But the offices themselves, and more particularly the old-fashioned class of directors, have much to answer for. To many of them the word "albuminuria," is like a lion in the path. Explanations and qualifications are of no avail to abate the force of their established convictions. They refuse even to consider the subject exactly as they would decline to hold a parley with a hungry lion. But is this wise? There are many cases in which it is as easy to decide that albuminuria is of a temporary and comparatively harmless character as it is to determine whether a cough is of phthisical origin or merely due to a common cold. The subject has much wider bearings than those of medical prejudices or life office profits. The whole question of thrift and provision for the future among all classes of the people is involved in it. There are a great many assurance offices, and a large volume of business is transacted by them. But if statistics of the business of all the offices were available for the outside world, most of us would be astonished at the comparatively small proportion of the adult male and female population who hold insurance policies.

Several of the leading physicians of this country have endeavoured, in the medical journals and elsewhere, to enlighten at least the general medical mind on the subject. But the whole population is as much entitled, and needs as much to be made familiar with the facts we are here considering, as it needs to be thoroughly instructed in the elements of house sanitation. Thrift is not a doctor's question only, nor a class question of any sort. It is a national and universal obligation; and he who, from prejudice or other cause, places impediments in the way of thriftiness, is a public enemy. We urge upon our professional brethren, and also upon the active managers of life offices, that duty and self-interest alike demand of them to spread abroad the truer and more scientific views of Bright's Disease, as well as of other vital conditions, that are rapidly becoming prevalent among instructed medical men.

